# Use of high cost care among Veterans with comorbid mental illness and Alzheimer’s Disease and related dementias

**DOI:** 10.1371/journal.pone.0282071

**Published:** 2023-05-12

**Authors:** Megan Shepherd-Banigan, Katherine E. M. Miller, S. Nicole Hastings, Loren J. Schleiden, Joshua M. Thorpe

**Affiliations:** 1 Durham VA Health Care System, Durham, NC, United States of America; 2 Department of Population Health Sciences, Duke University, Durham, NC, United States of America; 3 Duke-Margolis Center for Health Policy, Durham, NC, United States of America; 4 Hopkins Economics of Alzheimer’s Disease and Services Center, Johns Hopkins Bloomberg School of Public Health, Baltimore, Maryland, United States of America; 5 Division of Medical Ethics and Health Policy, Perelman School of Medicine at the University of Pennsylvania, Philadelphia, PA, United States of America; 6 Leonard Davis Institute of Health Economics, University of Pennsylvania, Philadelphia, PA, United States of America; 7 Geriatric Research, Education, and Clinical Center, Durham Veterans Affairs Health Care System, Durham, NC, United States of America; 8 Center for the Study of Aging, Duke University School of Medicine, Durham, NC, United States of America; 9 Division of Geriatrics, Department of Medicine, Duke University School of Medicine, Durham, NC, United States of America; 10 Center for Health Equity Research and Promotion, Veterans Affairs Pittsburgh Healthcare System, Pittsburgh, PA, United States of America; 11 Division of Pharmaceutical Outcomes and Policy, Eshelman School of Pharmacy, University of North Carolina, Chapel Hill, NC, United States of America; University of Toronto, CANADA

## Abstract

**Introduction/Objective:**

Alzheimer’s Disease and Other Related Dementias (AD/ADRD) leads to frequent emergency department (ED) and inpatient use. Mental health symptoms among persons with AD/ADRD increases cognitive and functional disabilities and could contribute to these high rates of intensive health care use. The objective of this paper is to assess the relationship of mental illness on 12-month patterns in hospitalization and ED use among Veterans aged 65 and over with a new AD/ADRD diagnosis.

**Methods:**

We used an existing dataset of administrative electronic health record data of Veterans with AD/ADRD from the US Veterans Health Administration linked with Medicare claims data from 2011–2015. We use multivariable logistic regression to examine the association between no pre-existing mental illness, pre-existing mental illness (e.g., major depressive disorder, generalized anxiety disorder, or post-traumatic stress disorder), and pre-existing severe mental illness—or SMI—(e.g., bipolar disorder, major depressive disorder with psychosis, or schizophrenia) and 12- month ED and hospitalization use and readmissions among Veterans who had an initial hospitalization visit. We estimated predicted probabilities, differential effect, and associated 95% confidence intervals.

**Results:**

In our sample, 1.4% had SMI and 11% had non-SMI mental illness. The unadjusted percentage with inpatient and ED use was higher among Veterans with SMI (34% and 26%, respectively) and Veterans with non-SMI mental illness (20%, 16%) compared with Veterans without pre-existing mental illness (12%, 9%). Compared to individuals with no pre-existing mental illness, having a pre-existing mental illness (1.27 percentage points, 95% CI: 0.76, 1.78) and a pre-existing SMI (7.17 percentage points, 95% CI: 5.66, 8.69) were both associated with an increased likelihood of ED use. The same pattern was observed for any inpatient use (mental illness 2.18, 95% CI: 1.59, 2.77; SMI 9.91, 95% CI: 8.21, 11.61). Only pre-existing SMI was associated higher hospitalization readmission.

**Discussion:**

Pre-existing mental illness increases use of high cost, intensive health care and this association is higher of more severe mental health conditions. We also show that pre-existing mental illness exerts a unique influence, above and beyond other comorbidities, such as diabetes, on ED and inpatient visits. More needs to be done to increase recognition of the unique risks of this combination of health conditions and encourage strategies to address them. Developing, testing, and implementing comprehensive strategies that address the intersection of ADRD and mental illness is promising approach that requires more focused attention.

## 1. Introduction

By 2060, the prevalence of Alzheimer’s Disease and Other Related Dementias (AD/ADRD) in the United States is expected to grow from 6.2 to 13.8 million [1]. In the absence of effective prevention or cures for AD/ADRD, high quality care is essential to preserve health-related quality of life. However, comorbid conditions, such as mental illness, present significant challenges to providing high quality care.

Addressing mental illness in older adults could be a high-impact approach to improve health-related quality of life among this population. Mental illness is a risk factor for cognitive decline and is prevalent in older adults with AD/ADRD [2, 3]. Globally, individuals with dementia have a 42% higher risk for hospitalization than those without AD/ADRD [4]. Persons with AD/ADRD are more frequent ED users; for example, in one study, the odds of having a 30-day repeat ED visit were 2.3 times higher among individuals with AD/ADRD, and survival rates were lower after an ED visit [5]. Hospitalizations among individuals with AD/ADRD may also be associated with greater ADL limitations, behavioral disturbances, and comorbidities. cognitive impairment [6]. For people with AD/ADRD, psychiatric symptoms and comorbidities increased cognitive and functional disabilities [2, 7] suggesting that these individuals with AD/ADRD and comorbid mental illness may be at even higher risk for inpatient and ED use than individuals without mental illness. However, to our knowledge, there are no recent studies that examine how mental illness affects healthcare utilization in ways that potentially worsen AD/ADRD risk and outcomes. We surmise that having a comorbid mental illness likely inhibits AD/ADRD identification and which leads to inappropriate clinical management because cognitive symptoms are erroneously attributed to mental illness [8]. Mental illness is also associated with poor health status [9–11]; the complexity of managing multiple health conditions can lead to fragmented care [12] and consequent medication-related safety risks [13].

The objective of this study is to examine 12-month health care use patterns among older Veterans with a new AD/ADRD diagnosis and a pre-existing mental illness (e.g., MDD, GAD, PTSD) and/or a serious mental illness (SMI)). We hypothesize that pre-existing mental illness will increase risk for any hospitalization, 30-day admission, and emergency department (ED) visit in the 12 months after AD/ADRD diagnosis. If pre-existing mental illness is associated with intensive, high cost care, we can invest in strategies to improve proactive treatment and management of mental illness in outpatient settings to decrease costs and improve health-related quality of life for older adults with AD/ADRD.

## 2. Methods

We examine the association between having diagnosed mental illness prior to incident AD/ADRD diagnosis and health care use in the year post-incident AD/ADRD diagnosis using regression analyses. We use data from the Veterans Health Administration (VHA) because mental illness is routinely screened in primary care and, therefore, we expect that ascertainment of mental health diagnoses in VHA administrative data will be more accurate than in administrative data from other health systems.

### 2.1. Data and sample

We use administrative claims data for care provided by the VHA and care in the community purchased by the VHA (VHA-purchased care) available in the VHA Corporate Data Warehouse (CDW) from 2009–2015 [14]. We also use claims and assessment data for care purchased by the Centers for Medicare & Medicaid (CMS) for Medicare Fee-for-service (FFS) beneficiaries for Fiscal Years 2011–2015. First, we use an existing VHA dataset constructed by Dr. Joshua Thorpe (IIR 12–379; VA Pittsburgh Healthcare System University Drive Division, Pittsburgh, PA; Funding Period: June 2014—April 2018). The data includes demographic, comorbidity, inpatient (including Medicare Provider Analysis and Review–MedPAR which contain 100% of Medicare beneficiaries hospital service use), outpatient, nursing home, vital status, and medication data from the VA Corporate Data Warehouse (CDW).

**Fig 1:**
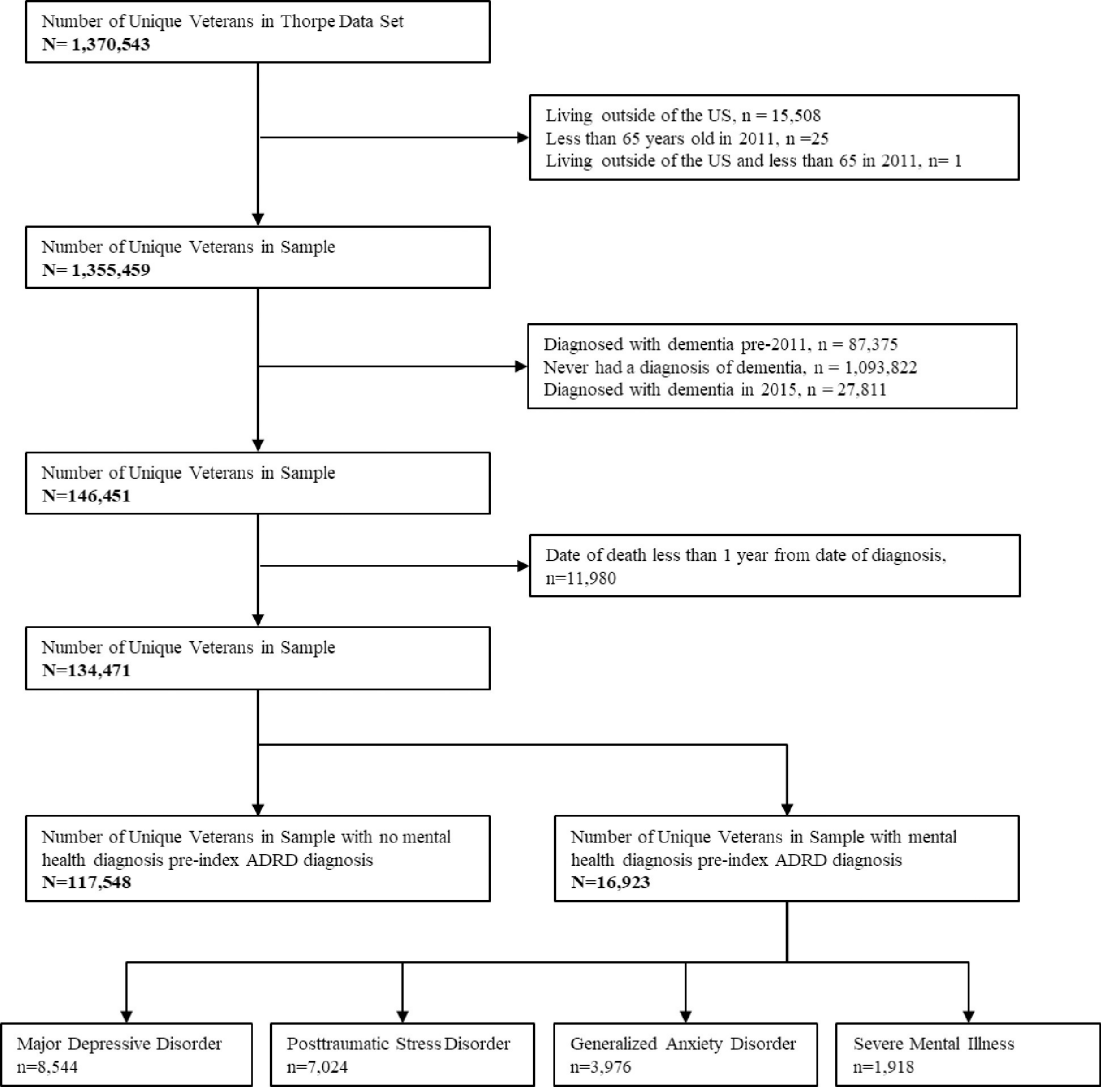
Study flow.

The sample includes Veterans aged 65 or older as of January 2011 with an incident AD/ADRD diagnosis between Fiscal Year (FY) 2011 and FY 2014 (see Fig 1 for study flow). We follow Veterans for 12 months post-incident diagnosis; therefore, the most recent cases of incident Alzheimer’s Disease and related dementias (AD/ADRD) occur in 2014 to allow for a 12 month follow up in 2015. Using criteria defined by Taylor et al., we define incident AD/ADRD as the first instance of an ICD-9-CM diagnosis code of 331.0; 331.1; 331.2; 331.7; 290.0; 290.1; 290.10; 290.11; 290.12; 290.13; 290.20; 290.21; 290.3; 290.40; 290.41; 290.42; 290.43; 294.0; 294.1; 294.8; or 797 in at least one inpatient, short-term nursing home, home health aide, hospital outpatient, or other outpatient visit identified through VA CDW and Medicare data between FY 2011–2014 [15]. We exclude Veterans if they 1) receive AD/ADRD diagnosis prior to 2011 or (2) died within 12 months post-incidence diagnosis. See Fig 1 for study flow.

### 2.2. Measures

#### 2.2.a. Outcomes

We examine each outcome for 12-months post-incidence AD/ADRD diagnosis.

*Any Emergency Department (ED) Use*. Using VA claims, we use stop codes [16] to identify emergency department visits not resulting in hospitalizations, as defined in Appendix A in S1 Appendix. Using Medicare FFS claims, we identify emergency visits not resulting in hospitalizations using outpatient and inpatient files with Revenue Codes 0450–0459, 0981 and inpatient MedPAR files with an emergency room charge amount greater than $0.

*Any Inpatient Use*. We identify inpatient care through (1) VHA inpatient census, acute care main file, extended care file, observation care; (2) VHA-purchased inpatient claims; and (3) MedPAR claims, excluding skilled nursing facility stays.

*Any 30-day Hospital Readmission*. We define having any 30-day hospital readmission as Veterans with inpatient use within 11 months post-incidence diagnosis and a subsequent inpatient use (for any reason) within 30 days of the first inpatient use as identified through either VA or Medicare data.

#### 2.2.b. Key explanatory variables

*Pre-existing Mental Illness*. We include an indicator variable of whether a Veteran had a diagnosis of major depressive disorder (MDD), posttraumatic stress disorder (PTSD), or generalized anxiety disorder (GAD) in the two years prior to incident AD/ADRD diagnosis (hereafter referred to as a pre-existing mental illness). We define diagnosed MDD as one or more diagnosis of ICD-9-CM: 296.2 or 296.3 except for 296.24 and 296.34. We define diagnosed PTSD as one or more diagnosis of ICD-9-CM: 309.81. We define diagnosed GAD as one or more diagnosis of ICD-9-CM: 300.02. Notably, MDD, PTSD, and GAD diagnoses are not mutually exclusive.

*Pre-existing Severe Mental Illness*. We include an indicator variable of whether a Veteran had a diagnosis of bipolar disorder, major depressive disorder with psychosis, or schizophrenia prior to incident AD/ADRD diagnosis (hereafter referred to as a pre-existing severe mental illness). We define use the presence of at least one of the following diagnosis codes: 296.7X, 295.0X, 295.7X, 298.0X, 296.24, or 296.34. All Veterans with pre-existing severe mental illness also had comorbid pre-existing mental illness as defined as major depressive disorder, posttraumatic stress disorder, and/or generalized anxiety disorder [17].

#### 2.2.c. Baseline covariates for adjustment

We include indicators of Veteran characteristics, including age, gender, marital status, race, ethnicity, any primary care use prior to incident post-AD/ADRD diagnosis, and priority enrollment group which categorizes Veteran copay requirements for VHA services and is defined by military service, disability, income, and other VA benefits (e.g., pension, disability compensation). To capture Veteran’s physical health, we include (1) indicators for diagnoses of myocardial infarction, benign and malignant neoplasms, diabetes, headache, hearing loss, hyperlipidemia, hypertension, and obesity and (2) the number of physical comorbidities. All covariates were indexed to the Veterans’ initial AD/ADRD diagnosis.

### 2.3. Statistical analysis

#### 2.3.a. Primary analysis

Using multiple multivariable logistic regressions, we examine the differences in probability of having any ED use or any inpatient use in the year post incidence AD/ADRD diagnosis among Veterans aged 65+ with pre-existing mental illness compared to Veterans aged 65+ without pre-existing mental illness. We examine the probability of any 30-day readmission post-incident AD/ADRD diagnosis conditional on having any inpatient use post-incident AD/ADRD diagnosis. All models include covariates described in section 2.2.c. (except for number of physical comorbidities due to multicollinearity) and with priority enrollment groups interacted with the three level indicator for: (1) no pre-existing mental illness or severe mental illness, (2) pre-existing mental illness only, and (3) pre-existing severe mental illness. Significant additional covariate interactions to improve model fit were assessed and included if statistically significant. We calculate predicted probabilities and the differential effect of having pre-existing mental illness and pre-existing severe mental illness for each outcome with an accepted significance level of 0.05.

#### 2.3.b. Secondary analysis

While the primary analysis adjusts for several indicators of Veterans’ physical health, unobserved differences between individuals with and without mental illness likely persist. For example, Veterans with an incident diagnosis of AD/ADRD and pre-existing mental illness may have higher health care use patterns. Thus, as a secondary analysis, we compare older Veterans with pre-existing mental illness to older Veterans without pre-existing mental illness but a diagnosis of a health condition associated with high health care use patterns. Specifically, we examine whether individuals with a pre-existing mental illness have differential health care use post-incident AD/ADRD diagnosis compared to individuals without a pre-existing mental illness or pre-existing severe mental illness but with a physical illness associated with greater health care use. We compare individuals with pre-existing mental illness or severe mental illness and no diagnosis of diabetes compared to Veterans with no diagnosed mental illnesses and diagnosis(es) of diabetes [18]. We model each outcome in this subsample using the same model specifications described in section 2.2.c. with covariates from section 2.3. See subsample description in Appendix B in S1 Appendix.

#### 2.3.c. Sensitivity analysis

As a sensitivity analysis, we conducted all models requiring at least two diagnoses for indicators of pre-existing mental illness and pre-existing severe mental illness.

All analyses were conducted using StataMP, version 17(College Station, TX: StataCorp LLC). The Durham VA and Pittsburgh VA institutional IRBs approved the study and waiver of consent prior to study activities (IRB# 1630568).

## 3. Results

### 3.1. Descriptive results

Table 1 presents the descriptive statistics of the overall sample of Veterans with AD/ADRD; those with pre-existing severe MI; those with a pre-existing MI; and, those without a pre-existing MI. For the sample of Veterans with AD/ADRD, the average age is 81 (SD 7), mostly male (93.4%), married (62.9%), white (86.8%), not Hispanic/Latino(a) (81.6%), a priority enrollment group 5–8 (65.4%), with an average of 1.8 physical health comorbidities. In the two years prior to AD/ADRD diagnosis, Veterans had diagnoses of hypertension (53.7%), hyperlipidemia (47.4%); hearing loss (26.4%); diabetes (24.1%); neoplasm (20.4%); obesity (7.7%); myocardial infarction (3.0%); and, headache (2.9%). In the two years prior to AD/ADRD diagnosis, nearly 69% of Veterans with AD/ADRD had any primary care use; 28.5% had any ED use; and, 12.5% had any inpatient uses. Among this study sample, Veterans with pre-existing mental illness or severe mental illness are younger at incident diagnosis and a higher proportion are female, not-married, not-white, Hispanic/Latino(a), and in priority enrollment group 1. Veterans with pre-existing mental illness or severe mental illness also have worse physical health as indicated by number of physical health comorbidities and as a higher proportion of Veterans with pre-existing mental illness or severe mental illness are diagnosed across most physical health comorbidities than Veterans without pre-existing mental illness. Veterans with pre-existing mental illness or severe mental illness also have more VHA primary care use, ED use, and hospitalization use in the two years prior to incident AD/ADRD diagnosis.

**Table 1:** Descriptive statistics of overall sample with AD/ADRD and by any pre-existing mental health diagnosis.

	Overall Sample N = 134,471	Pre-existing Severe Mental illness N = 1,918	Pre-existing Mental Illness N = 15,005	No Pre-existing Mental Illness N = 117,548
**Demographic Characteristics**				
Age at time of AD/ADRD diagnosis, mean (SD)	81.223 (7.066)	75.051 (6.469)	78.678 (7.835)	81.649 (6.854)
Gender, %				
Female	3313 (2.5%)	82 (4.3%)	384 (2.6%)	2847 (2.4%)
Male	125554 (93.4%)	1778 (92.7%)	13891 (92.6%)	109885 (93.5%)
Missing	5604 (4.2%)	58 (3.0%)	730 (4.9%)	4816 (4.1%)
Marital Status, %				
Divorced, Widowed, or Separated	28960 (21.5%)	602 (31.4%)	3242 (21.6%)	25116 (21.4%)
Married	84542 (62.9%)	911 (47.5%)	9432 (62.9%)	74199 (63.1%)
Never Married	6338 (4.7%)	300 (15.6%)	847 (5.6%)	5191 (4.4%)
Missing	14631 (10.9%)	105 (5.5%)	1484 (9.9%)	13042 (11.1%)
Race, %				
White	116702 (86.8%)	1627 (84.8%)	12856 (85.7%)	102219 (87.0%)
Black or African American	13658 (10.2%)	242 (12.6%)	1601 (10.7%)	11815 (10.1%)
Other	3494 (2.6%)	35 (1.8%)	448 (3.0%)	3011 (2.6%)
Missing	617 (0.5%)	14 (0.7%)	100 (0.7%)	503 (0.4%)
Ethnicity, %				
Hispanic or Latino	3371 (2.5%)	66 (3.4%)	570 (3.8%)	2735 (2.3%)
Not Hispanic or Latino	109662 (81.6%)	1668 (87.0%)	12330 (82.2%)	95664 (81.4%)
Unknown by patient	1852 (1.4%)	27 (1.4%)	227 (1.5%)	1598 (1.4%)
Declined to answer	2125 (1.6%)	24 (1.3%)	239 (1.6%)	1862 (1.6%)
Missing	17461 (13.0%)	133 (6.9%)	1639 (10.9%)	15689 (13.3%)
Priority Enrollment Group, %				
Group 1	14115 (10.5%)	472 (24.6%)	3962 (26.4%)	9681 (8.2%)
Group 2	6492 (4.8%)	101 (5.3%)	1156 (7.7%)	5235 (4.5%)
Group 3	12744 (9.5%)	132 (6.9%)	1516 (10.1%)	11096 (9.4%)
Group 4	5838 (4.3%)	75 (3.9%)	378 (2.5%)	5385 (4.6%)
Group 5	39717 (29.5%)	688 (35.9%)	3642 (24.3%)	35387 (30.1%)
Group 6	2389 (1.8%)	28 (1.5%)	329 (2.2%)	2032 (1.7%)
Group 7	21291 (15.8%)	175 (9.1%)	1463 (9.8%)	19653 (16.7%)
Group 8	24600 (18.3%)	168 (8.8%)	1655 (11.0%)	22777 (19.4%)
Missing	7285 (5.4%)	79 (4.1%)	904 (6.0%)	6302 (5.4%)
**Physical Health**				
Number of comorbidities, mean (SD)	1.9 (1.63)	2.6 (1.59)	2.5 (1.7)	1.8 (1.6)
Myocardial Infarction, n (%)	4027 (3.0%)	103 (5.4%)	774 (5.2%)	3150 (2.7%)
Benign or Malignant Neoplasm, n (%)	27435 (20.4%)	545 (28.4%)	4257 (28.4%)	22633 (19.3%)
Diabetes, n (%)	32397 (24.1%)	692 (36.1%)	5090 (33.9%)	26615 (22.6%)
Headache, n (%)	3912 (2.9%)	160 (8.3%)	1020 (6.8%)	2732 (2.3%)
Hearing loss, n (%)	35548 (26.4%)	522 (27.2%)	5052 (33.7%)	29974 (25.5%)
Hyperlipidemia, n (%)	63695 (47.4%)	1229 (64.1%)	8915 (59.4%)	53551 (45.6%)
Hypertension, n (%)	72163 (53.7%)	1341 (69.9%)	10062 (67.1%)	60760 (51.7%)
Obesity, n (%)	10397 (7.7%)	362 (18.9%)	2146 (14.3%)	7889 (6.7%)
**Health Care Use**				
Any VHA Primary Care Use in 2 years prior to AD/ADRD diagnosis, %	92146 (68.5%)	1629 (84.9%)	12053 (80.3%)	78464 (66.8%)
Number of VHA primary care visits in 2 years prior to AD/ADRD diagnosis, median (IQR)	2.000 (0.000, 6.000)	5.000 (2.000, 10.000)	5.000 (1.000, 9.000)	2.000 (0.000, 6.000)
Any Emergency Department Visits in 2 years prior to AD/ADRD diagnosis, %	38321 (28.5%)	1004 (52.3%)	6564 (43.7%)	30753 (26.2%)
Number of Emergency Department visits in 2 years prior to AD/ADRD diagnosis, median (IQR)	2.000 (1.000, 3.000)	2.000 (1.000, 5.000)	2.000 (1.000, 4.000)	2.000 (1.000, 3.000)
Any Inpatient Use in 2 years prior to AD/ADRD diagnosis, %	16831 (12.5%)	857 (44.7%)	3541 (23.6%)	12433 (10.6%)
Number of Hospitalizations in 2 years prior to AD/ADRD diagnosis, median (IQR)	1.000 (1.000, 3.000)	2.000 (1.000, 3.000)	2.000 (1.000, 3.000)	1.000 (1.000, 2.000)

NOTES: Any pre-existing mental health illness diagnoses include major depressive disorder, posttraumatic stress disorder, generalized anxiety disorder. Severe mental illness diagnoses include bipolar disorder, major depressive disorder with psychosis, and/or schizophrenia. Veterans who died within 1 year of diagnosis have already been excluded from the sample. Priority enrollment refers to the level of healthcare coverage a Veteran has from VHA. A more comprehensive definition is in Section 2.2.c. and Group 1 affords the most health care coverage (i.e., the lowest copay) and Group 8 the least health care coverage.

Table 2 presents the descriptive statistics of Veterans by type of pre-existing mental illness. As Veterans can be represented across multiple diagnoses, indicators of statistical significance are not presented. Demographic characteristics and health care use trends prior to incident AD/ADRD diagnosis are consistent and similar across all diagnoses. Notably, a greater proportion of Veterans diagnosed with PTSD are in priority enrollment group 1 and have physical health diagnoses, most notably hypertension, hyperlipidemia, and diabetes.

**Table 2:** Descriptive statistics by pre-existing mental illness.

	Major Depressive Disorder N = 8,544	Posttraumatic Stress Disorder N = 7,024	Generalized Anxiety Disorder N = 3,976
**Demographic Characteristics**			
Age at time of AD/ADRD diagnosis, mean (SD)	78.381 (7.506)	77.144 (8.222)	79.736 (7.527)
Gender, %			
Female	279 (3.3%)	98 (1.4%)	142 (3.6%)
Male	7667 (89.7%)	6900 (98.2%)	3471 (87.3%)
Missing	598 (7.0%)	26 (0.4%)	363 (9.1%)
Marital Status, %			
Divorced, Widowed, or Separated	1959 (22.9%)	1479 (21.1%)	889 (22.4%)
Married	4886 (57.2%)	5009 (71.3%)	2182 (54.9%)
Never Married	545 (6.4%)	446 (6.3%)	239 (6.0%)
Missing	1154 (13.5%)	90 (1.3%)	666 (16.8%)
Race, %			
White	7566 (88.6%)	5569 (79.3%)	3654 (91.9%)
Black or African American	727 (8.5%)	1102 (15.7%)	225 (5.7%)
Other	190 (2.2%)	293 (4.2%)	76 (1.9%)
Missing	61 (0.7%)	60 (0.9%)	21 (0.5%)
Ethnicity, %			
Hispanic or Latino	292 (3.4%)	383 (5.5%)	94 (2.4%)
Not Hispanic or Latino	6704 (78.5%)	6284 (89.5%)	3071 (77.2%)
Unknown by patient	127 (1.5%)	112 (1.6%)	58 (1.5%)
Declined to answer	146 (1.7%)	105 (1.5%)	54 (1.4%)
Missing	1275 (14.9%)	140 (2.0%)	699 (17.6%)
Priority Enrollment Group, %			
Group 1	1428 (16.7%)	3236 (46.1%)	622 (15.6%)
Group 2	485 (5.7%)	790 (11.2%)	221 (5.6%)
Group 3	771 (9.0%)	811 (11.5%)	381 (9.6%)
Group 4	281 (3.3%)	78 (1.1%)	122 (3.1%)
Group 5	2487 (29.1%)	1174 (16.7%)	1094 (27.5%)
Group 6	155 (1.8%)	236 (3.4%)	50 (1.3%)
Group 7	1022 (12.0%)	358 (5.1%)	461 (11.6%)
Group 8	1184 (13.9%)	306 (4.4%)	586 (14.7%)
Missing	731 (8.6%)	35 (0.5%)	439 (11.0%)
**Physical Health**			
Number of comorbidities, mean (SD)	2.31 (1.8)	3.03 (1.5)	2.03 (1.7)
Myocardial Infarction, n (%)	2214 (25.9%)	442 (6.3%)	160 (4.0%)
Benign or Malignant Neoplasm, n (%)	2727 (31.9%)	2439 (34.7%)	905 (22.8%)
Diabetes, n (%)	585 (6.8%)	2937 (41.8%)	1013 (25.5%)
Headache, n (%)	2531 (29.6%)	625 (8.9%)	231 (5.8%)
Hearing loss, n (%)	4738 (55.5%)	2993 (42.6%)	1046 (26.3%)
Hyperlipidemia, n (%)	5271 (61.7%)	4992 (71.1%)	2011 (50.6%)
Hypertension, n (%)	1203 (14.1%)	5608 (79.8%)	2247 (56.5%)
Obesity, n (%)	2214 (25.9%)	1263 (18.0%)	444 (11.2%)
**Health Care Use**			
Any Primary Care Use in 2 years prior to AD/ADRD diagnosis, %	6321 (74.0%)	6641 (94.5%)	2775 (69.8%)
Number of primary care visits in 2 years prior to AD/ADRD diagnosis, median (IQR)	4.000 (0.000, 9.000)	6.000 (4.000, 11.000)	3.000 (0.000, 8.000)
Any Emergency Department Visits in 2 years prior to AD/ADRD diagnosis, %	4223 (49.4%)	2750 (39.2%)	1999 (50.3%)
Number of Emergency Department visits in 2 years prior to AD/ADRD diagnosis, median (IQR)	2.000 (1.000, 4.000)	2.000 (1.000, 4.000)	2.000 (1.000, 4.000)
Any Inpatient Use in 2 years prior to AD/ADRD diagnosis, %	2245 (26.3%)	2082 (29.6%)	779 (19.6%)
Number of Hospitalizations in 2 years prior to AD/ADRD diagnosis, median (IQR)	2.000 (1.000, 3.000)	2.000 (1.000, 3.000)	2.000 (1.000, 3.000)

NOTES: Veterans who died within 1 year of diagnosis have already been excluded from the sample.

In the 12 months post-incident AD/ADRD diagnosis, on average, 10.3% of Veterans had any ED use and 13.6% had any inpatient use. Among Veterans that had any inpatient use post-incident AD/ADRD diagnosis, 10.2% had a readmission for any reason within 30-days, see Table 3. As these results are descriptive, we did not directly compare proportions across groups. Visual inspection of proportions suggests that compared to Veterans without pre-existing mental illness, a seemingly higher proportion of Veterans with pre-existing mental illness and severe mental illness had any ED use (15.8% and 25.9%, respectively, versus 9.3%); any inpatient use (20.4% and 34.4%, respectively, versus 12.3%); and 30-day readmission (12.0% and 16.4%, respectively, versus 9.6%) in the year post-incident AD/ADRD diagnosis. These trends appeared to be consistent across types of pre-existing mental illnesses, see Table 4.

**Table 3:** Descriptive statistics of outcomes post-incident AD/ADRD.

	Overall Sample N = 134,471	Pre-existing Severe Mental Illness N = 1,918	Pre-existing Mental Illness N = 15,005	No Pre-existing Mental Illness N = 117,548
Any Emergency Department Visit in 12 months post AD/ADRD diagnosis, %	13814 (10.3%)	496 (25.9%)	2368 (15.8%)	10950 (9.3%)
Any Inpatient Use in 12 months post AD/ADRD diagnosis, ^a^ %	18233 (13.6%)	659 (34.4%)	3066 (20.4%)	14508 (12.3%)
30-Day Readmission in 12 months post AD/ADRD diagnosis conditional on having any post-diagnosis hospitalization, %	1863 (10.2%)	108 (16.4%)	368 (12.0%)	1387 (9.6%)

^a^ This does *not* include hospitalizations if the incident AD/ADRD diagnosis was received during a hospitalization.

NOTES: Any pre-existing mental health illness diagnoses include major depressive disorder, posttraumatic stress disorder, generalized anxiety disorder. Severe mental illness diagnoses include bipolar disorder, major depressive disorder with psychosis, and/or schizophrenia. For Veterans who died at least one year post AD/ADRD diagnosis. Veterans who died within 1 year of diagnosis have already been excluded from the sample.

**Table 4:** Descriptive statistics of outcomes post-incident AD/ADRD diagnosis.

	Major Depressive Disorder N = 8,544	Posttraumatic Stress Disorder N = 7,024	Generalized Anxiety Disorder N = 3,976
Any Emergency Department Visit in 12 months post AD/ADRD diagnosis, %	1355 (15.9%)	1413 (20.1%)	525 (13.2%)
Any Inpatient Use in 12 months post AD/ADRD diagnosis, ^a^ %	1760 (20.6%)	1831 (26.1%)	641 (16.1%)
30-Day Readmission in 12 months post AD/ADRD diagnosis conditional on having any post-diagnosis hospitalization, %	216 (12.3%)	229 (12.5%)	86 (13.4%)

^a^ This does *not* include hospitalizations if the incident AD/ADRD diagnosis was received during a hospitalization.

### 3.2. Association of pre-existing mental illness and outcomes

**Fig 2:**
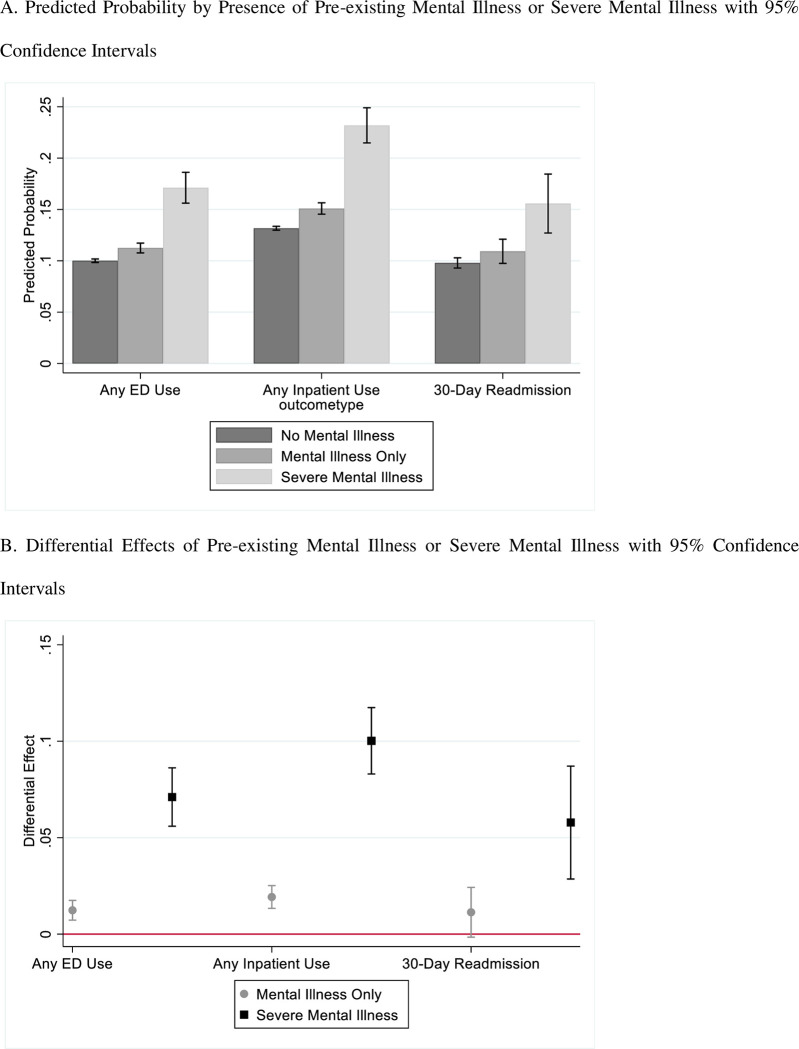
Predicted probability and differential effect from primary analysis. A. Predicted Probability by Presence of Pre-existing Mental Illness or Severe Mental Illness with 95% Confidence Intervals. B. Differential Effects of Pre-existing Mental Illness or Severe Mental Illness with 95% Confidence Intervals. NOTES: Fig 2A shows the predicted probability of each health care outcome among Veterans with (1) no diagnosed mental illness, (2) diagnosed mental illness only (major depressive disorder, posttraumatic stress disorder, generalized anxiety disorder), and (3) diagnosed with mental illness and severe mental illness (bipolar disorder, major depressive disorder with psychosis, and/or schizophrenia) prior to index AD/ADRD diagnosis. The red lines at the top of each bar correspond to the 95% confidence intervals. Fig 2B shows the differential effect of (1) diagnosed mental illness only (major depressive disorder, posttraumatic stress disorder, generalized anxiety disorder), and (2) diagnosed with mental illness and severe mental illness (bipolar disorder, major depressive disorder with psychosis, and/or schizophrenia) compared to having no diagnosed mental illness or severe mental illness prior to index AD/ADRD diagnosis. For example, Veterans with no diagnosed mental illness, diagnosed mental illness only, and diagnosed with mental illness and severe mental illness have a 0.10, 0.11, and 0.17 predicted probability of having an ED visit in the year post AD/ADRD incident diagnosis, respectively. Correspondingly, compared to Veterans with no diagnosed mental illness, Veterans diagnosed mental illness only and diagnosed with mental illness and severe mental illness are 1.2 and 7.1 percentage points more likely to have an ED visit in the year post AD/ADRD incident diagnosis, respectively (p<0.05).

We find having a pre-existing mental illness (major depressive disorder, posttraumatic stress disorder, and/or generalized anxiety disorder) is associated with increased probability of any ED use, inpatient use, and 30-day readmissions compared with Veterans without a pre-existing mental illness (see Fig 2). Having a pre-existing mental illness is associated with a 1.24 percentage point increase in the probability of having any ED use post-incident AD/ADRD diagnosis (95% CI: 0.72, 1.75), controlling for all variables in the model. Having a pre-existing severe mental illness (bipolar disorder, major depressive disorder with psychosis, or schizophrenia) is associated with a 7.11 percentage point increase in probability of having any ED use post-incident AD/ADRD diagnosis (95% CI: 5.60, 8.62). When examining any inpatient use post-incident AD/ADRD diagnosis, we find having a pre-existing mental illness and pre-existing severe mental illness is associated with a 1.92 and 10.02 percentage point increase, respectively, in the probability of having any inpatient use post-incident AD/ADRD diagnosis (95% CI: 1.33, 2.51 and 8.30, 11.74, respectively), controlling for all variables in the model. Among Veterans with any inpatient use 11 months post-incident AD/ADRD diagnosis, we find no evidence of having a pre-existing mental illness is associated with the probability of 30-day readmission (p = 0.086) while having pre-existing severe mental illness is associated with 5.78 percentage point increase in probability of 30-day readmission (95% CI: 2.86, 8.71).

### 3.3. Secondary analysis

**Fig 3:**
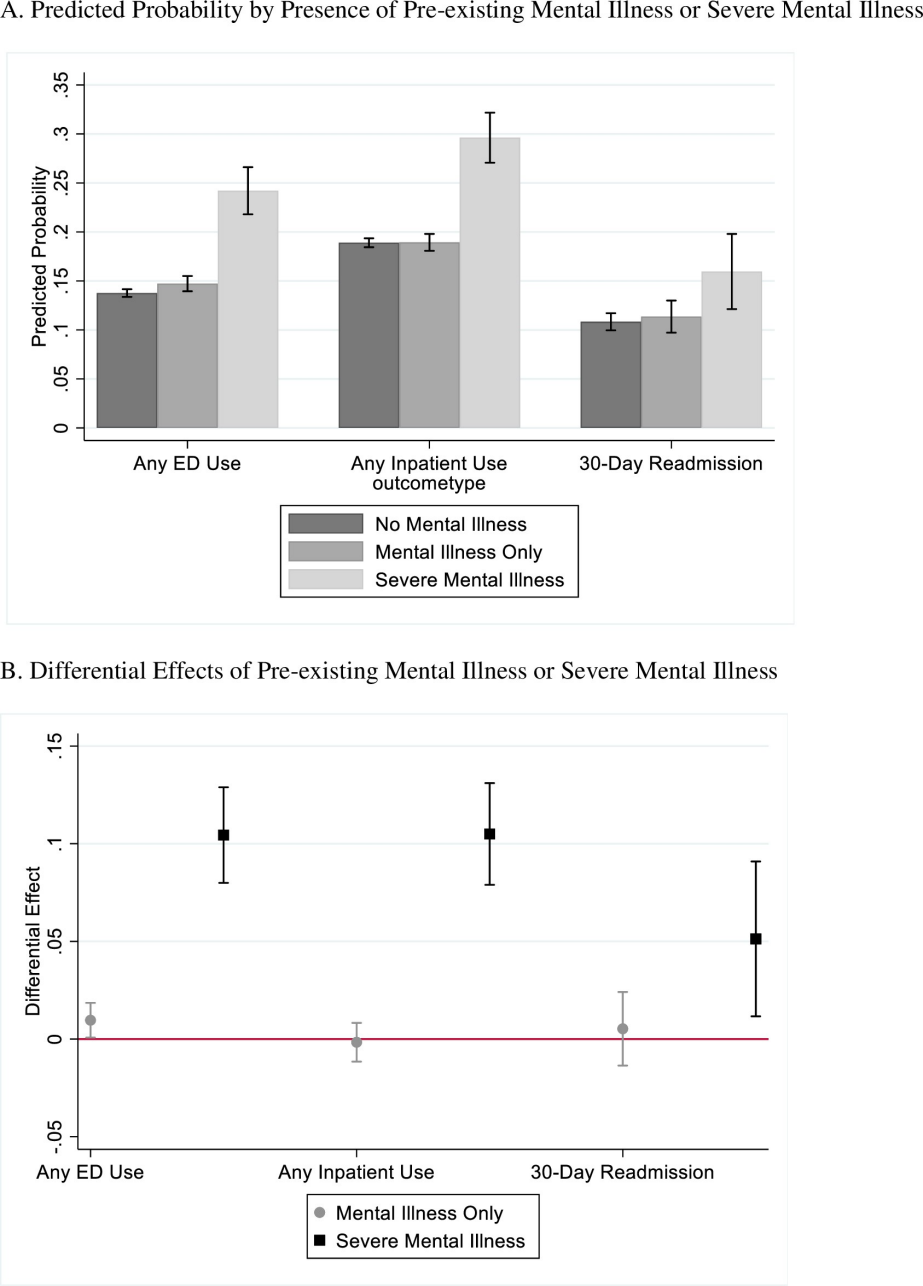
Predicted probability and differential effect from secondary analysis. A. Predicted Probability by Presence of Pre-existing Mental Illness or Severe Mental Illness. B. Differential Effects of Pre-existing Mental Illness or Severe Mental Illness. NOTES: Fig 3A shows the predicted probability of each health care outcome among Veterans with (1) the comparison group (no diagnosed mental illness or severe mental illness and diagnosed diabetes), (2) diagnosed mental illness only (major depressive disorder, posttraumatic stress disorder, generalized anxiety disorder) and no diagnosis of diabetes, and (3) diagnosed with mental illness and severe mental illness (bipolar disorder, major depressive disorder with psychosis, and/or schizophrenia) and no diagnosis of diabetes prior to index AD/ADRD diagnosis. The red lines at the top of each bar correspond to the 95% confidence intervals. Fig 3B shows the differential effect of (1) diagnosed mental illness only (major depressive disorder, posttraumatic stress disorder, generalized anxiety disorder), and (2) diagnosed with mental illness and severe mental illness (bipolar disorder, major depressive disorder with psychosis, and/or schizophrenia) compared to Veterans without diagnosed mental illness or severe mental illness and with diagnosis of diabetes prior to index AD/ADRD diagnosis. For example, Veterans with no diagnosed mental illness, diagnosed mental illness only, and diagnosed with mental illness and severe mental illness have a 0.13, 0.14, 0.24 predicted probabilities of having an ED visit in the year post AD/ADRD incident diagnosis, respectively. Correspondingly, compared to Veterans with no diagnosed mental illness, Veterans diagnosed mental illness only and diagnosed with mental illness and severe mental illness are 0.96 and 10.44 percentage points more likely to have an ED visit in the year post AD/ADRD incident diagnosis (p<0.05).

Comparing Veterans with no pre-existing mental illness, no pre-existing severe mental illness, and a diagnosis of diabetes prior to incident AD/ADRD diagnosis (as described in section 2.2.b), we find having a pre-existing mental illness is associated with increased probability of any ED use, as aligned with the primary analysis (see Fig 3). We also find having a pre-existing severe mental illness is associated with increased probability of any ED use, inpatient use, or 30-day hospital readmission (p<0.05).

Results of the sensitivity analysis were consistent with the primary analysis. See S1 Appendix for full model results.

## 4. Discussion

We found that pre-existing serious mental illness was associated with a higher risk for any ED use, any inpatient use, and 30-day readmission while pre-existing mental illness was associated with a statistical significant higher risk for any ED visit and inpatient use only. Two studies conducted 20+ years ago found that older Veterans with AD/ADRD and a co-existing psychiatric condition had more inpatient visits than older Veterans with depression only [19] or AD/ADRD only [20]. Veterans with AD/ADRD and depression may have received suboptimal care as these Veterans also received fewer medical and psychiatric outpatient visits than patients with depression only [19]. Our study reexamines this question using recent VHA data among general mental illness and extends past research to examine the effects in specific mental health subgroups. We also found that the association between pre-existing mental illness and service use persisted even when we restricted the comparison group to individual with diabetes, another complex comorbidity. Our findings suggest that risk for high cost, intensive health care may increase as the complexity of the mental illness increases.

### 4.1. Strengths and limitations

We used data from a large administrative dataset of Veteran healthcare use. While administrative data is limited by the potential for unmeasured confounding and measurement error, VHA data is a rich source of longitudinal data that should have a high capture rate of mental illness given that Veterans are routinely screened for mental illness. To maximize correct classification of mental illness versus AD/ADRD, we used validated definitions of AD/ADRD and mental health diagnoses. We also define mental illness as a diagnosis that appeared within the two years prior to AD/ADRD to minimize capturing mental health symptoms that are part of the dementia syndrome. Despite this effort, mental health symptoms may appear as part of prodromal dementia up to 10 years before AD/ADRD is actually diagnosed [21] and therefore misclassification might still exist for MDD and GAD. We also excluded individuals who died before the 12 months observation period to ensure that we had complete follow up on all study subjects. We suspect that Veterans who were excluded due to death may have been sicker and that might have had a higher likelihood of ED and inpatient use due to comorbid AD/ADRD and mental illness; therefore, we expect that any bias in our estimates due to this exclusion would have been towards the null. To check the robustness of our findings we also examined the association between PTSD and SMI and the outcomes as these conditions both have well-defined diagnostic criteria that are distinct from AD/ADRD. The trends in results for each of these diagnoses were similar to those of MDD and GAD which suggests that mental illness, more broadly, increases high cost, intensive health service use. Finally, to explore the extent to which mental illness exerts a unique influence on inpatient and ED use compared with other comorbidities, we conducted a sensitivity analysis in which we restricted our comparison group to have diabetes diagnosis and found that pre-existing mental illness increased ED and inpatient use.

### 4.2 Implications

The implications of these results are that more needs to be done to increase recognition of the unique risks of this combination of health conditions and encourage strategies to address them. At the patient level, health care providers should assess and treat mental health symptoms in older adults with AD/ADRD [8, 22]. However, it is notable that both AD/ADRD and mental illness are complex conditions in older adults. Mental illness is associated with many other physical health comorbidities, such as diabetes and cardiovascular disease [9–11]—which are also risk factors for AD/ADRD [23]. Therefore, integrated health system strategies may be needed. For example, improving system-wide care coordination for older adults with multiple complex health conditions could improve outcomes [24]. Care coordination could involve increasing systematic interactions and communications among various providers through collaborative care or case management models.

While existing models that target care for older adults with AD/ADRD are promising [25–31], they have not been disseminated broadly [12] and are currently not the standard of care. Elements of these models include medical home models targeting AD/ADRD and depression [26], embedding memory clinics within primary care [32], and team-based care management that includes a dementia specialist [31]. Furthermore, some of these models do not focus on the intersection between AD/ADRD and mental illness. While mental illness is one of many potential AD/ADRD comorbidities, mental illness likely presents unique challenges given the overlap in symptoms with AD/ADRD. Also, mental illness substantially increases functional decline in older adults with dementia [33] and older adults with mental illness may experience less support from family [34] to help them engage in care. Therefore, interventions for AD/ADRD may need to specifically address the unique health and social needs associated with comorbid mental illness.

### 4.3 Conclusions

We have evidence to suggest that among older adults with AD/ADRD, pre-existing mental illness, particularly SMI, is a risk factor for costly care. Yet, healthcare teams are at a loss for how to diagnose and manage these conditions because systems of care for mental health and ADRD run separately with minimal coordination [25, 35]. Future research is needed to test models of care to diagnose, treat, and support individuals with AD/ADRD and comorbid mental illness and their families.

## Supporting information

S1 Appendix Contains supporting tables. (DOCX)
