# Supplementary material for: Use of high cost care among Veterans with comorbid mental illness and Alzheimer’s Disease and related dementias
Source: PLoS One. 2023 May 12;18(5):e0282071. doi: 10.1371/journal.pone.0282071 (PMC10180599; doi:10.1371/journal.pone.0282071)
Supplement: S1 Appendix — (DOCX) [file pone.0282071.s001.docx]

Appendix A

We identify ED visits using the clinic stop code 130 or 131 with any secondary stop code excluding those listed below in table A1.

Table A1. Excluded Secondary Stop Codes

| 107 | ELECTROCARDIOGRAM (EKG) | 329 | MEDICAL PROCEDURE UNIT |
| --- | --- | --- | --- |
| 115 | ULTRASOUND | 334 | CARDIAC STRESS TEST/EXERCISE TOLERANCE TEST (ETT) |
| 152 | ANGIOGRAM CATHETERIZATION | 430 | CYSTO ROOM IN UROLOGY CLINIC |
| 311 | PACEMAKER | 435 | SURGICAL PROCEDURE UNIT |
| 321 | GASTROINTESTINAL (GI) ENDOSCOPY | 474 | RESEARCH |
| 328 | MEDICAL SURGICAL DAY UNIT (MSDU) | 999 | EMPLOYEE HEALTH |

<https://www.va.gov/OPTOMETRY/docs/VHA_Directive_2011-022_Copayment_for_Outpatient_Medical_Care_Provided_to_Veterans_by_DVA.pdf>

Appendix B. Descriptive Statistics of Sub-Sample used for Secondary Analysis

|  | Any Pre-existing Severe Mental Illness ^a^ without Diabetes Diagnosis  N=1226 | Any Pre-existing Mental Illness ^b^ without Diabetes Diagnosis  N= 9915 | Any Pre-existing Diabetes Diagnosis without any Pre-existing Mental Illness  N = 26615 |
| --- | --- | --- | --- |
| Age at time of AD/ADRD diagnosis, mean (SD) | 75.626 (6.600) | 79.573 (7.856) | 80.449 (6.653) |
|  |  |  |  |
| Gender, % |  |  |  |
| Female | 58 (4.7%) | 292 (2.9%) | 458 (1.7%) |
| Male | 1110 (90.5%) | 8893 (89.7%) | 26155 (98.3%) |
| Missing | 58 (4.7%) | 730 (7.4%) | 2 (<1%) |
|  |  |  |  |
| Marital Status, % |  |  |  |
| Divorced, Widowed, or Separated | 380 (31.0%) | 2075 (20.9%) | 6207 (23.3%) |
| Married | 536 (43.7%) | 5808 (58.6%) | 18978 (71.3%) |
| Missing | 104 (8.5%) | 1465 (14.8%) | 87 (0.3%) |
| Never Married | 206 (16.8%) | 567 (5.7%) | 1343 (5.0%) |
|  |  |  |  |
| Race, % |  |  |  |
| White | 1075 (87.7%) | 8721 (88.0%) | 22120 (83.1%) |
| Black or African American | 117 (9.5%) | 871 (8.8%) | 3617 (13.6%) |
| Other or Multiple Races, Declined, or Missing | 34 (2.7%) | 323 (3.3%) | 878 (3.3%) |
|  |  |  |  |
| Ethnicity, % |  |  |  |
| Hispanic or Latino | 37 (3.0%) | 287 (2.9%) | 952 (3.6%) |
| Missing | 127 (10.4%) | 1575 (15.9%) | 713 (2.7%) |
| Not Hispanic or Latino | 1029 (83.9%) | 7743 (78.1%) | 24123 (90.6%) |
| Declined to answer or Unknown by patient | 33 (2.7%) | 310 (3.1%) | 827 (3.1%) |
|  |  |  |  |
| Priority Enrollment Group, % |  |  |  |
| Group 1 | 252 (20.6%) | 2231 (22.5%) | 3140 (11.8%) |
| Group 2 | 55 (4.5%) | 654 (6.6%) | 1451 (5.5%) |
| Group 3 | 77 (6.3%) | 982 (9.9%) | 2564 (9.6%) |
| Group 4 | 56 (4.6%) | 251 (2.5%) | 1065 (4.0%) |
| Group 5 | 463 (37.8%) | 2398 (24.2%) | 9327 (35.0%) |
| Group 6 | 18 (1.5%) | 222 (2.2%) | 324 (1.2%) |
| Group 7 | 113 (9.2%) | 1064 (10.7%) | 4115 (15.5%) |
| Group 8 | 114 (9.3%) | 1209 (12.2%) | 4610 (17.3%) |
| Missing/None | 78 (6.4%) | 904 (9.1%) | 19 (0.1%) |
|  |  |  |  |
| Any VHA Primary Care Use in 2 years prior to AD/ADRD diagnosis, % | 963 (78.5%) | 7087 (71.5%) | 25890 (97.3%) |
| Number of VHA primary care visits in 2 years prior to AD/ADRD diagnosis, median (IQR) | 4.000 (1.000, 9.000) | 4.000 (0.000, 7.000) | 5.000 (3.000, 9.000) |
|  |  |  |  |
| Any Emergency Department Visits in 2 years prior to AD/ADRD diagnosis, % | 639 (52.1%) | 4267 (43.0%) | 8434 (31.7%) |
| Number of Emergency Department visits in 2 years prior to AD/ADRD diagnosis, median (IQR) | 2.000 (1.000, 4.000) | 2.000 (1.000, 4.000) | 2.000 (1.000, 3.000) |
|  |  |  |  |
| Any Inpatient Use in 2 years prior to AD/ADRD diagnosis, % | 483 (39.4%) | 1793 (18.1%) | 5517 (20.7%) |
| Number of Hospitalizations in 2 years prior to AD/ADRD diagnosis, median (IQR) | 2.000 (1.000, 3.000) | 2.000 (1.000, 3.000) | 2.000 (1.000, 3.000) |
|  |  |  |  |
| Physical Health Comorbidity |  |  |  |
| Number of comorbidities, mean (SD) | 1.9 (1.4) | 1.8 (1.5) | 3.5 (1.1) |
| Myocardial Infarction, n (%) | 62 (5.1%) | 374 (3.8%) | 1374 (5.2%) |
| Benign or Malignant Neoplasm, n (%) | 333 (27.2%) | 2439 (24.6%) | 7810 (29.3%) |
| Diabetes, n (%) | 0 (0.0%) | 0 (0.0%) | 26615 (100.0%) |
| Headache, n (%) | 94 (7.7%) | 588 (5.9%) | 1021 (3.8%) |
| Hearing loss, n (%) | 310 (25.3%) | 3074 (31.0%) | 8907 (33.5%) |
| Hyperlipidemia, n (%) | 654 (53.3%) | 4751 (47.9%) | 20972 (78.8%) |
| Hypertension, n (%) | 738 (60.2%) | 5461 (55.1%) | 23275 (87.5%) |
| Obesity, n (%) | 150 (12.2%) | 816 (8.2%) | 4449 (16.7%) |

^a^ Any pre-existing severe mental illness is defined as bipolar disorder, major depressive disorder with psychosis, or schizophrenia.

^b^ Any pre-existing mental health diagnosis is defined as Major Depressive Disorder (MDD), Posttraumatic stress disorder (PTSD), and/or Generalized Anxiety Disorder (GAD).

Appendix C. Primary Model Output with Odds Ratios and 95% CI

|  | Emergency Department Use | | Hospitalization | | 30-day Readmission | | |
| --- | --- | --- | --- | --- | --- | --- | --- |
|  | Odds ratio | (95% Confidence Interval) | Odds ratio | (95% Confidence Interval) | Odds ratio | (95% Confidence Interval) | |
| No Mental Illness | -ref- |  | -ref- |  | -ref- | |  |
| Mental Illness | 0.997 | 0.903, 1.102 | 1.012 | 0.926, 1.107 | 1.158 | | 0.915,1.466 |
| Severe Mental Illness | 1.358 | 1.083, 1.704 | 1.779 | 1.446, 2.188 | 1.468 | | 0.963,2.238 |
|  |  |  |  |  |  | |  |
| Priority enrollment group | 0.929 | 0.920, 0.938 | 0.892 | 0.885, 0.900 | 0.971 | | 0.945,0.997 |
|  |  |  |  |  |  | |  |
| No Mental Illness x Priority enrollment group | -ref- |  | -ref- |  | -ref- | |  |
| Mental Illness x Priority enrollment group | 1.032 | 1.010, 1.055 | 1.037 | 1.016, 1.057 | 0.994 | | 0.941,1.051 |
| Severe Mental Illness x Priority enrollment group | 1.084 | 1.033, 1.138 | 1.044 | 0.998, 1.092 | 1.035 | | 0.943,1.137 |
|  |  |  |  |  |  | |  |
| Age at time of diagnosis | 0.943 | 0.895, 0.994 | 0.866 | 0.827, 0.906 | 0.997701 | | 0.991,1.005 |
|  |  |  |  |  |  | |  |
| Female | -ref- |  | -ref- |  | -ref- | |  |
| Male | 1.077 | 0.955, 1.215 | 1.070 | 0.963, 1.190 | 1.116 | | 0.816,1.525 |
| Missing | 0.051 | 0.012, 0.202 | 0.041 | 0.010, 0.1641 | 1 | |  |
|  |  |  |  |  |  | |  |
| Marital Status |  |  |  |  |  | |  |
| Divorced, Widowed, or Separated | -ref- |  | -ref- |  | -ref- | |  |
| Married | 0.625 | 0.599, 0.651 | 0.595 | 0.573, 0.618 | 0.881 | | 0.793,0.979 |
| Missing | 0.157 | 0.126, 0.194 | 0.112 | 0.091, 0.137 | 0.957 | | 0.478,1.915 |
| Never Married | 1.006 | 0.932, 1.085 | 1.141 | 1.067, 1.221 | 0.809 | | 0.670,0.977 |
|  |  |  |  |  |  | |  |
| White race | 0.588 | 0.561, 0.616 | 0.680 | 0.651, 0.711 | 1.036 | | 0.916,1.172 |
| Not Hispanic | -ref- |  | -ref- |  | -ref- | |  |
| Hispanic | 2.097 | 1.908, 2.305 | 2.116 | 1.953, 2.293 | 1.020 | | 0.787,1.320 |
| Missing | 2.717 | 2.378, 3.103 | 2.342 | 2.079, 2.638 | 0.891 | | 0.619,1.283 |
|  |  |  |  |  |  | |  |
| Number of primary care visits | 1.056 | 1.053, 1.059 | 1.049 | 1.047, 1.052 | 1.021 | | 1.008,1.035 |
|  |  |  |  |  |  | |  |
| Myocardial Infarction | 1.692 | 1.562, 1.832 | 2.080 | 1.935, 2.237 | 1.292 | | 1.095,1.526 |
| Benign or Malignant Neoplasm | 1.560 | 1.498, 1.625 | 1.414 | 1.363, 1.468 | 1.106 | | 0.997,1.227 |
| Diabetes | 1.035 | 0.992, 1.081 | 1.202 | 1.157, 1.249 | 1.098 | | 0.985,1.223 |
| Headache | 1.815 | 1.679, 1.962 | 1.401 | 1.298, 1.512 | 1.253 | | 1.048,1.498 |
| Hearing loss | 1.070 | 1.027, 1.115 | 0.934 | 0.900, 0.970 | 0.948 | | 0.850,1.056 |
| Hyperlipidemia | 0.924 | 0.884, 0.966 | 0.866 | 0.832, 0.901 | 0.928 | | 0.826,1.043 |
| Hypertension | 1.474 | 1.402, 1.550 | 1.343 | 1.285, 1.403 | 1.053 | | 0.917,1.208 |
| Obesity | 1.139 | 1.074, 1.208 | 1.117 | 1.058, 1.179 | 1.068 | | 0.927,1.231 |
|  |  |  |  |  |  | |  |
| Age at baseline x Age at baseline | 1.000 | 1.000, 1.001 | 1.001 | 1.001, 1.001 | 1.00 | | 0.999,1.000 |
|  |  |  |  |  |  | |  |
| Constant | 1.252 | 0.154, 10.178 | 55.743 | 8.864, 350.570 | 0.111 | | 0.053,0.230 |

Appendix D. Sensitivity Analysis Model Output with Odds Ratios and 95% CI

|  | Emergency Department Use | | Hospitalization | | 30-day Readmission | | |
| --- | --- | --- | --- | --- | --- | --- | --- |
|  | Odds ratio | (95% Confidence Interval) | Odds ratio | (95% Confidence Interval) | Odds ratio | (95% Confidence Interval) | |
| No Mental Illness | -ref- |  | -ref- |  | -ref- | |  |
| Mental Illness | 1.130 | 0.979, 1.304 | 0.990 | 0.871, 1.125 | 1.063 | | 0.755, 1.496 |
| Severe Mental Illness | 1.681 | 1.237, 2.285 | 1.873 | 1.412, 2.485 | 1.676 | | 0.941, 2.986 |
|  |  |  |  |  |  | |  |
| Priority enrollment group | 0.946 | 0.931, 0.962 | 0.904 | 0.891, 0.917 | 0.962 | | 0.922, 1.002 |
|  |  |  |  |  |  | |  |
| No Mental Illness x Priority enrollment group | -ref- |  | -ref- |  | -ref- | |  |
| Mental Illness x Priority enrollment group | 0.992 | 0.962, 1.023 | 1.000 | 0.972, 1.028 | 0.998 | | 0.921, 1.081 |
| Severe Mental Illness x Priority enrollment group | 1.055 | 0.990, 1.124 | 1.002 | 0.944, 1.064 | 0.984 | | 0.863, 1.121 |
|  |  |  |  |  |  | |  |
| Age at time of diagnosis | 0.900 | 0.823, 0.983 | 0.848 | 0.783, 0.917 | 1.077 | | 0.865, 1.342 |
|  |  |  |  |  |  | |  |
| Female | -ref- |  | -ref- |  | -ref- | |  |
| Male | 1.021 | 0.832, 1.254 | 1.206 | 0.997, 1.459 | 1.270 | | 0.736, 2.194 |
| Missing | 0.087 | 0.012, 0.644 | 1.000 |  |  | |  |
|  |  |  |  |  |  | |  |
| Marital Status |  |  |  |  |  | |  |
| Divorced, Widowed, or Separated | -ref- |  | -ref- |  | -ref- | |  |
| Married | 0.712 | 0.665, 0.763 | 0.647 | 0.609, 0.689 | 0.897 | | 0.761, 1.057 |
| Missing | 0.353 | 0.232, 0.537 | 0.225 | 0.148, 0.343 | 1.221 | | 0.358, 4.159 |
| Never Married | 1.011 | 0.893, 1.144 | 1.227 | 1.100, 1.369 | 0.881 | | 0.666, 1.166 |
|  |  |  |  |  |  | |  |
| White race | 0.560 | 0.520, 0.603 | 0.708 | 0.661, 0.759 | 1.138 | | 0.942, 1.374 |
|  |  |  |  |  |  | |  |
| Missing | -ref- |  | -ref- |  | -ref- | |  |
| Not Hispanic | 1.941 | 1.642, 2.296 | 2.061 | 1.778, 2.389 | 0.920 | | 0.605, 1.400 |
| Hispanic | 2.533 | 2.037, 3.149 | 2.147 | 1.759, 2.620 | 0.847 | | 0.485, 1.478 |
|  |  |  |  |  |  | |  |
| Number of primary care visits | 1.053 | 1.049, 1.057 | 1.047 | 1.043, 1.051 | 1.008 | | 1.000, 1.016 |
|  |  |  |  |  |  | |  |
| Myocardial Infarction | 1.580 | 1.406, 1.775 | 1.953 | 1.757, 2.171 | 1.320 | | 1.044, 1.670 |
| Benign or Malignant Neoplasm | 1.491 | 1.400, 1.588 | 1.366 | 1.290, 1.447 | 1.019 | | 0.873, 1.188 |
| Headache | 1.890 | 1.686, 2.118 | 1.491 | 1.334, 1.666 | 1.364 | | 1.065, 1.747 |
| Hearing loss | 1.071 | 1.003, 1.143 | 0.954 | 0.899, 1.011 | 0.953 | | 0.810, 1.121 |
| Hyperlipidemia | 0.969 | 0.899, 1.044 | 0.948 | 0.887, 1.013 | 0.879 | | 0.735, 1.051 |
| Hypertension | 1.540 | 1.395, 1.699 | 1.480 | 1.359, 1.612 | 1.290 | | 0.997, 1.669 |
| Obesity | 1.157 | 1.067, 1.255 | 1.144 | 1.062, 1.231 | 1.184 | | 0.982, 1.429 |
|  |  |  |  |  |  | |  |
| Age at baseline x Age at baseline | 1.001 | 1.000, 1.001 | 1.001 | 1.001, 1.002 | 1.000 | | 0.998, 1.001 |
|  |  |  |  |  |  | |  |
| Constant | 7.504 | 0.224, 251.862 | 104.862 | 4.585, 2398.343 | 0.005 | | 0.000, 30.743 |
